# Rupture bilatérale spontanée et simultanée du tendon quadricipital dans l’adénome parathyroïdien: à propos d’un cas et revue de la littérature

**DOI:** 10.11604/pamj.2018.29.14.13540

**Published:** 2018-01-04

**Authors:** Amour Espoir Mokoko-Louckou, Badarou Chaibou, Ismail Abdouli, Kevin Parfait Bienvenu Bouhelo-Pam, Mohammed El Idrissi, Mohammed Shimi, Abdelhalim El Ibrahimi, Abdelmajid El Mrini

**Affiliations:** 1 Service de Traumato-Orthopédie B4, CHU Hassan II, Fès, Maroc

**Keywords:** Adénome parathyroïdien, genou, rupture spontanée, tendon quadricipal, Parathyroid adenoma, knee, spontaneous rupture, quadriceps tendon

## Abstract

La rupture spontanée du tendon quadricipital est rare, ainsi qu'une atteinte bilatérale et simultanée. Nous rapportons un patient de 53 ans suivi pour adénome parathyroïdien compliqué d'insuffisance rénale, admis pour rupture bilatérale et spontanée des deux tendons quadricipitaux. Il a bénéficié d'une réparation chirurgicale qui lui a permis une autonomie.

## Abstract (en)

Spontaneous as well as bilateral and simultaneous rupture of the quadriceps tendon is rare. We report the case of a 53-year old patient followed up for parathyroid adenoma complicated by renal failure, admitted for spontaneous bilateral rupture of the two quadriceps tendons. He underwent surgical repair allowing him to maintain his autonomy.

## Introduction

Les ruptures spontanées du tendon quadricipital sont rares. Elles surviennent selon un mécanisme indirect par contracture de l'appareil extenseur [1]. Lorsqu'il n'y a pas de notion de traumatisme, il faut rechercher une maladie systémique pouvant fragiliser les tendons [2]. Nous rapportons le cas d'un patient présentant une rupture bilatérale spontanée et simultanée du tendon quadricipital.

## Patient et observation

**Figure 1:**
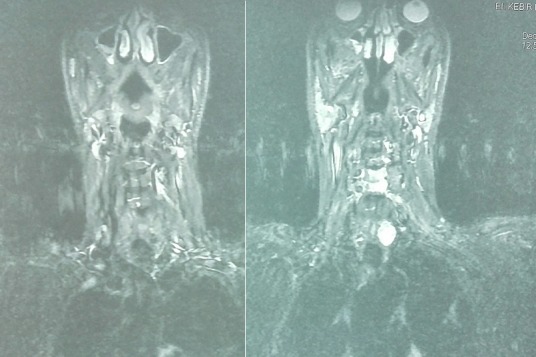
IRM du cou

**Figure 2:**
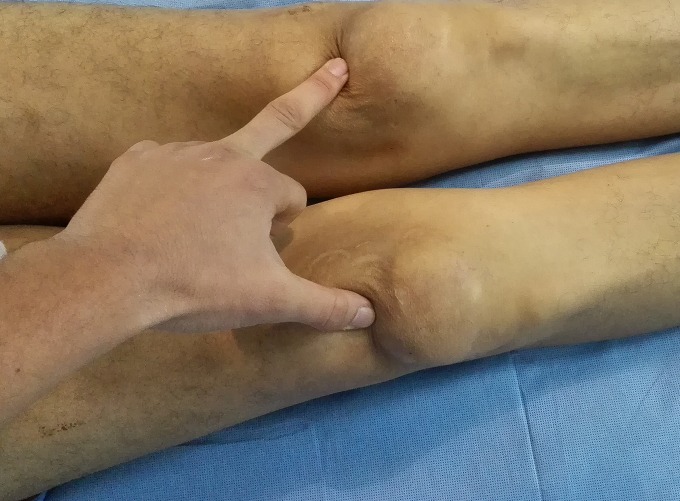
Image Clinique

**Figure 3:**
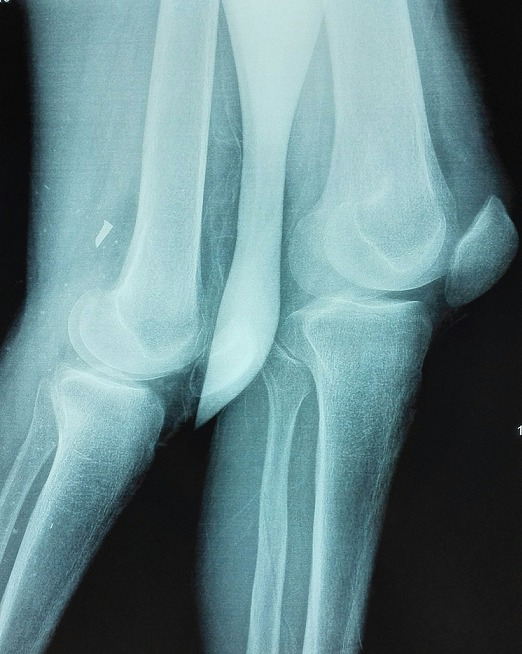
Image radiologique

**Figure 4:**
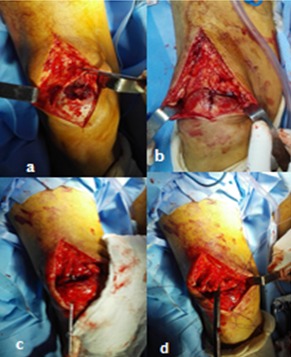
(A) rupture du tendon quadricipital droit; (B) rupture du tendon quadricipital gauche; (C) point trans osseux s’appuyant sur la rotule; (D) réparation chirurgicale

Il s'agissait d'un patient âgé de 53 ans, arabe, suivi pour une insuffisance rénale chronique et une hypercalcémie secondaire à l'adénome parathyroïdien (Figure 1), admis dans notre formation pour prise en charge de douleurs aux deux genoux avec impotence fonctionnelle totale des deux membres inférieurs constatées par le patient lors du lever matinal. Le patient était autonome avant cette symptomatologie, avec un indice de masse corporelle à 20,36 kg/m^2^. L'examen clinique avait objectivé une dépression supra-patellaire bilatérale (Figure 2), douloureuse à la palpation avec déficit d'extension active des deux genoux. On ne notait pas d'ouverture cutanée ni d'atteinte vasculo-nerveuse. La radiographie standard de profil des deux genoux montre un abaissement des deux rotules sans fracture associée (Figure 3). L'échographie des deux cuisses et genoux avait objectivé les ruptures complètes des tendons quadricipitaux, à environ 1 cm de son insertion patellaire à droite et 2 cm à gauche. Après un bilan préopératoire complet, le patient était admis au bloc, sous locorégionale, en décubitus dorsal, par voie d'abord antérieure médiane longitudinale centrée sur le bord supérieur de la rotule pour les deux genoux. A l'exploration, on avait noté un hématome sous facial mimine bilatéral, avec rupture complète des fibres tendineuses à bord déchiqueté, à 1cm de l'insertion rotulienne à droite et 2 cm à gauche (Figure 4 A et B). Nous avions procédé à l'évacuation de l'hémarthrose, lavage au sérum physiologique, avivement des berges puis suture bord à bord par des points en « U » avec des points trans-osseux s'appuyant sur la rotule (Figure 4 C et D) et fermeture sur un drain aspiratif. Les deux genoux étaient immobilisés par des attelles genouillères pendant 3 semaines avec des soins locaux 1jour sur 2 et ablation des agrafes après 21 jours. Le patient avait bénéficié d'une rééducation fonctionnelle (à but de renforcement musculaire et amélioration des amplitudes articulaires).Nous avions évalué le résultat fonctionnel par le score KSCRS (Knee society clinical rating system) qui permet d'apprécier la douleur, la mobilité et la force musculaire [3]. Il était bon à 3 mois (70à droit et 72 à gauche) excellent à 6 mois (97 des deux côtés).

## Discussion

L'hyperparathyroïdie primaire est une affection fréquente, son traitement est chirurgicale par l'exérèse de l'adénome parathyroïdien [4]. Plusieurs études ont montré les manifestations musculo-squelettiques dans l'hyperparathyroïdie primaire [5]. Les ruptures spontanées et bilatérales simultanées du tendon quadricipital sont essentiellement rencontrées dans le cadre d'une pathologie fragilisant les tendons [6,7]. Dans notre étude, il s'agissait d'un adénome parathyroïdien [8].

## Conclusion

Les ruptures bilatérales spontanées et simultanées du tendon quadricipital sont exceptionnelles. Une pathologie fragilisant les tendons reste la principale cause. Dans plusieurs études nous avons retrouvé une hyperparathyroïdie primaire quelques soit la cause, mais dans notre cas la cause était un adénome parathyroïdien.

## Conflits d’intérêts

Les auteurs ne déclarent aucun conflit d'intérêts.
